# Evaluation of the effect of physical therapy on pain and dysfunction of knee osteoarthritis based on fNIRS: a randomized controlled trial protocol

**DOI:** 10.1186/s12891-022-06074-2

**Published:** 2023-02-28

**Authors:** Xiao-yi Wang, Chun-cha Bao, Ran An, Tao Wu, Dun Wang, Yu-jia Zhang, Cheng-qi He

**Affiliations:** 1 Department of Rehabilitation Medicine, West China Hospital, Sichuan University, Chengdu, 610041 Sichuan People’s Republic of China; 2 Key Laboratory of Rehabilitation Medicine, West China Hospital, Sichuan University, 610041 Chengdu, People’s Republic of China

**Keywords:** Knee osteoarthritis, Rehabilitation, Physical therapy, Functional near-infrared spectroscopy

## Abstract

**Background:**

Knee osteoarthritis (KOA) is a chronic musculoskeletal disease that can cause joint pain and dysfunction, affecting the quality of life of patients. Nonsurgical treatment is the conventional treatment of KOA, among which physical therapy is widely used because of its simplicity, convenience and effectiveness. The functional biomarker will add to the clinical fidelity and diagnostic accuracy. Therefore, our study chose a more objective evaluation indicator, functional near-infrared spectroscopy (fNIRS), to identify between healthy people and KOA patients, and to detect the pain change before and after treatment of KOA patients.

**Methods:**

The study will be conducted in the Rehabilitation Medical Center of West China Hospital of Sichuan University and divided into 2 stages. In the first stage, we will compare and determine the differences in baseline data between healthy volunteers and KOA patients. In the second stage, 72 KOA patients will be randomly divided into two groups: the drug therapy group (DT) and the combination therapy group (CT) for 10 treatments. Outcome measures will be measured at baseline and on the 5th and 10th days after the intervention, including the numerical rating scale (NRS), Western Ontario and McMaster Universities Osteoarthritis Index (WOMAC), pain catastrophizing scale (PCS), the association of pain severity with task-state functional connectivity fNIRS and association of pain severity with task-activated fNIRS.

**Discussion:**

By analyzing the fNIRS data of healthy volunteers and KOA patients, our study will be determined whether fNIRS can be used as a new indicator to reflect the severity of pain in KOA patients. Subsequently, the same fNIRS data for KOA patients before and after the intervention will be collected to provide an accurate evaluation criterion for the effect of physical therapy on KOA.

**Trial registration:**

The study was registered on the Chinese Registry website (registered in ChiCTR.org with the identifiers ChiCTR2200064175 and 29/09/2022).

## Background

Osteoarthritis (OA) is the most common degenerative joint disorder, causing chronic joint pain, physical function limitations and the reduction of quality of life (QoL) [1]. The main risk factors contributing to the initiation and progression of OA are aging, obesity and joint trauma [2, 3]. With the aging of the world, the prevalence of OA is gradually increasing, primarily focused on middle-aged and old people [2, 4]. Epidemiology investigations show that OA affects approximately 260 million people worldwide [5], accounting for 2.4% of the total number of disabled people in the world [2]. Knee osteoarthritis (KOA) is the most common type of OA. Musculoskeletal pain is the crucial symptom and primary reported outcome in KOA patients [6]. Furthermore, KOA pain is the major driver of functional limitations and the negative impact on quality of life [7]. Current KOA pain management therapies include nonpharmacological, pharmacological (NSAIDs), and surgical treatments [8]. Although drugs are effective for relieving pain, their analgesic capacity attenuates and causes adverse side effects over time [1].

To date, some clinical trials of treatments for KOA have demonstrated that physical therapy can effectively relieve pain and improve function, and also decreased the need for analgesic drugs [9, 10]. Recently, the combination of physical modalities has been widely discussed, such as low-intensity pulsed ultrasound (LIPUS) [11]. LIPUS is a physical therapy that is used to improve functional capacity in KOA [11, 12]. In addition, ultrashort wave therapy is also effective physiotherapy, which mainly depends on the thermal effect to relieve inflammation and pain in the knee joint [13].

In recent years, based on the development of neuroimaging techniques, studies on KOA disease-specific measurements have shown that many regions of the brain, such as the prefrontal cortex, the prefrontal lobe and the marginal area, are involved in the regulation of pain intensity [14]. Furthermore, Baliki et al. reported that the knee joint of KOA patients can activate the neural components of pain perception under pressure stimulation, and compared with the normal control group, the bilateral activity of the cerebral cortex and cingulate cortex of the patients increased significantly [15, 16]. Functional near-infrared spectroscopy (fNIRS) is a technology that quantifies cortical hemodynamics using noninvasive and portable means [17, 18]. The measurements in oxy- and deoxyhemoglobin concentrations represent the hemodynamic responses in the cerebral cortex [18]. FNIRS relies on the neurovascular mechanism of cerebral blood flow to record cerebral cortical activity. Respond to the activities of brain regions caused by spontaneous pain by recording the changes in hemoglobin concentration in the cerebral cortex [19]. The study summarized relevant brain regions in the fNIRS evaluation of pain state including Primary Motor Cortex (M1) (parietal, frontal, and temporal cortical regions) and Supplementary motor area (SMA) and so on [20]. Similarly, the results of the fNIRS evaluation of chronic pain (myofibrillary pain and back pain) showed motor cortex dysfunction in patients with pain [21]. A recent clinical study proved the feasibility of using fNIRS to evaluate the pain response in KOA patients and found that there was a certain correlation between the activation of the prefrontal cortex and pain in KOA patients [22]. Additionally, another study used fNIRS to explore the longitudinal effects of transcranial direct current stimulation (tDCS) on the hemodynamic response to pain in KOA patients and found that cortical activity increased over time [23]. Research on physical factor therapy based on fNIRS to improve the pain of KOA patients is still relatively limited. Therefore, our study introduces a new detection method to evaluate physical therapy for relieving pain and dysfunction in KOA patients and provides a new choice for the clinical evaluation of OA severity.

## Methods

**Fig. 1:**
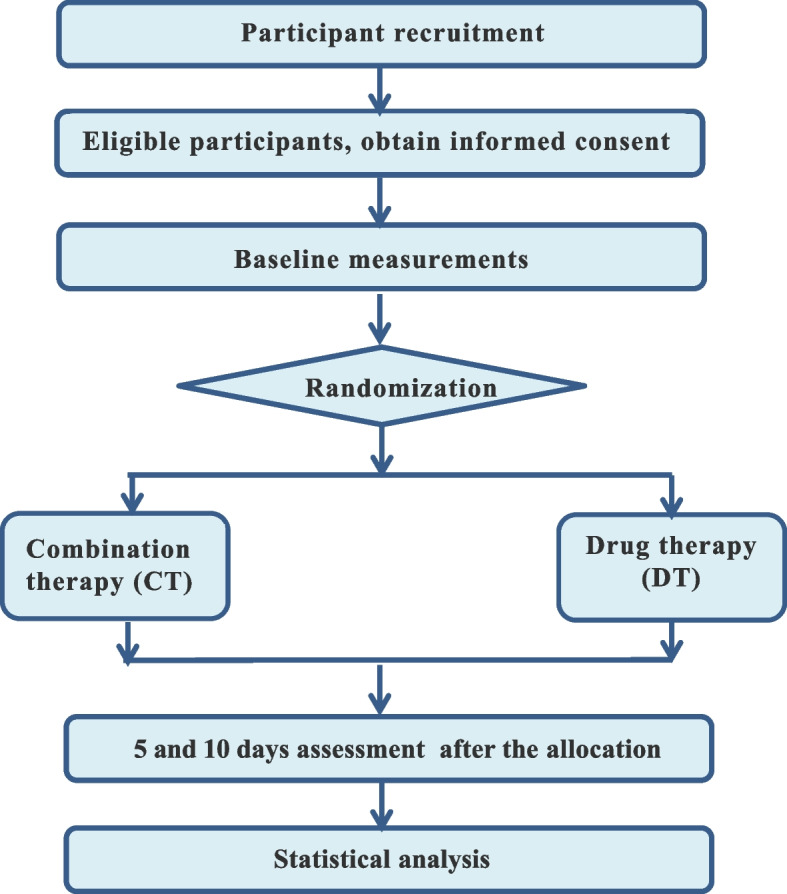
Flow chart of the second stage

The trial will be divided into two stages. The first stage is to analyze the baseline data of healthy volunteers and KOA patients and compare the differences between the two groups. In the second stage, the KOA patients will be randomly divided into two groups, and the outcome indicators will be analyzed after the intervention. This stage is a single-center, single-blinded, parallel randomized controlled trial (RCT). The trial will follow the Recommendations for Interventional Trials [24], and the flow diagram is shown in Fig. 1. The trial will be conducted in West China Hospital, Sichuan University, Chengdu, China. All participants will sign written informed consent forms.

### Participants

Two trained rehabilitation physicians will recruit 24 healthy volunteers over 50 years old and 72 KOA patients according to the inclusion and exclusion criteria. Patients should meet the following conditions: (1) diagnosed with KOA according to the 2021 Chinese Orthopedics Association diagnosis and treatment guidelines [25]; (2) knee pain (previous week) score ≥ 3 on the NRS; (3) Kellgren–Lawrence grade I-III [26]; (4) right-handed; (5) normal lower limbs; and (6) no open wounds or metal foreign bodies near the knee joint.

The exclusion criteria were as follows: (1) diagnosed with rheumatoid arthritis, gout or severe osteoporosis; (2) having undergone knee surgery or received an intra knee injection within the past 6 months; (3) experiencing depression (using the Self-rating depression scale, depression severity index < 0. 50 is no depression [27]), anxiety (using the Self-rating Anxiety Scale, standard score < 50 is no anxiety [28]), mania (using the Bech-Rafaelsen Mania Scale, ≤ 5 is no mania [29]), dementia and obvious cognitive impairment (using the Montreal Cognitive Assessment, ≥ 26 is normal [30]); (4) having a cancerous tumor, severe bronchiectasis, acute suppurative inflammation, high fever, active pulmonary tuberculosis, glaucoma, heart failure, severe anemia, cerebrovascular disease, or having a pacemaker implanted in the body; (5) having a history of epilepsy or taking antiepileptic drugs; (6) taking drugs that change the excitability of the cerebral cortex (such as sedative-hypnotics or antidepressants); (6) unable to participate in the trial due to other health problems; and (7) unable to sign the informed consent or participate in other scientific research projects(for example, a sudden fall, sudden low back pain, shoulder pain and other sudden diseases).

### Interventions

In the first stage, 24 healthy volunteers and 72 KOA patients will not receive any treatment, with only one baseline data measurement. In the second stage, 72 patients with KOA will be randomly divided into two groups: the drug therapy group (DT) and the combination therapy group (CT).

#### Drug therapy group (DT)

*Education*: The patients will receive a doctor's oral education after grouping, including disease introduction, common rehabilitation treatment methods, weight management, emotional management, and daily exercise-related precautions.

*Drug*: All patients should take glucosamine hydrochloride capsules and celecoxib capsules orally [25].

#### Combination therapy group (CT)

The combination therapy group included the same education and drug as the DT, along with 10 sessions of physical therapy.

*Physical therapy*: patients will receive 2 courses of physical therapy, including 10 sessions of ultrashort wave therapy (Shantou Medical Equipment Factory Co., Ltd., China) and LIPUS (Shenzhen Dongdixin Technology Co., Ltd., China). Ultrashort wave therapy takes 20 min for each knee joint, and the treatment intensity is based on the patient's feelings, that is, there is a feeling of warmth during treatment. LIPUS takes 5 min for each knee joint, and the treatment intensity is 1.4 W/cm^2^. All treatments will be performed by two trained physiotherapists to ensure therapeutic efficacy.

During the trial, patients’ condition is aggravated, and they can be allowed to use relevant drugs for treatment. However, any medication use will be recorded in the medical record and diary.

### Outcome measures

Outcome measures will be measured according to Osteoarthritis Research Society International [31]. Baseline measurements will be performed in the first stage, and the second stage will be measured at baseline and 5 (T5) and 10 (T10) days after intervention. The timeline for the second stage is shown in Table 1.

**Table 1:**
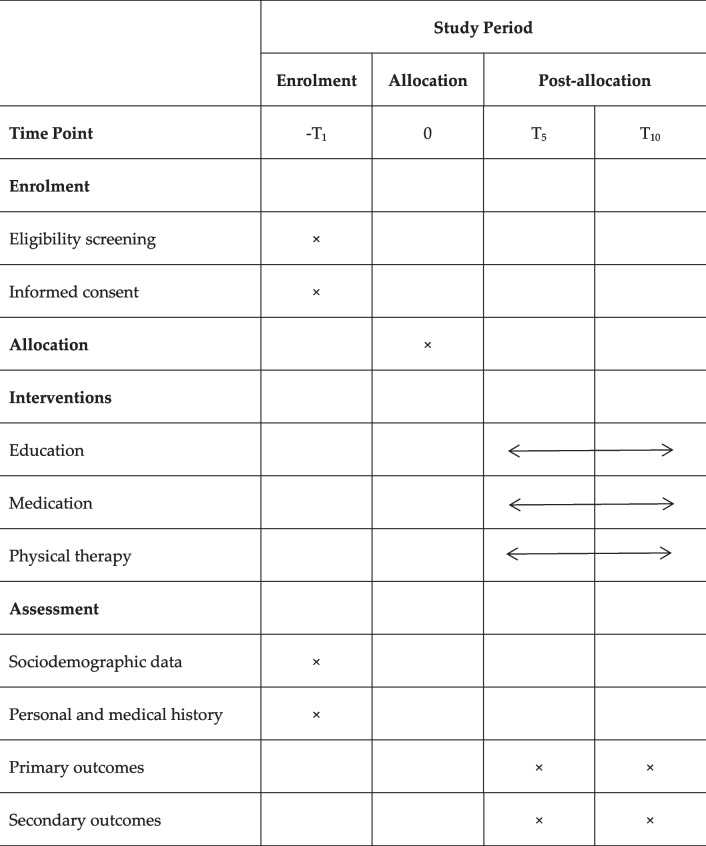
The participant timeline in the second stage

### Primary outcomes:

- (1) The difference in knee pain before and after treatment, will be measured by the NRS, a standard tool for the study of chronic pain, that is an 11-number scale from 0 to 10, with 0 being no pain and 10 being severe pain. The minimum clinically important difference (MICD) is 1.8 [32, 33].
- (2) Association of pain severity with task-activated fNIRS. Data collection: The NirSmart portable near-infrared functional brain imaging system (Danyang Huichuang Medical Equipment Co., Ltd., China) will collect the cerebral cortical activity of the participants in the 30-s chair-stand test, the timed up-and-go test, the 40-m fast-paced walk test and the stair-climb test (9 steps) [34]. The test will be carried out in a quiet corridor and stairwell. Before each test, the test procedure will be explained to the participants, and the participants will be informed that if there is no special situation to keep quiet during the test, the test will be started after the participants fully understand. At the beginning of each test, the participants will need to stand quietly for 10 s to obtain the resting state fNIRS, and then the corresponding test will be carried out according to the rules. Forty effective test channels consisting of 24 light transmitters and 16 light receivers cover the functional areas of the whole brain. Changes in blood oxygen will be measured at two wavelengths (760 and 850 nm) with a sampling frequency of 10 Hz.

### Secondary outcomes

- (1) The Western Ontario and McMaster Universities Osteoarthritis Index (WOMAC) will be used to assess knee pain, stiffness and physical function. The WOMAC is a self-assessed health status scale for patients with KOA consisting of 24 items in three subscales of pain (5 items), stiffness (2 items), and physical function (17 items). All items are scored from 0 (asymptomatic) to 4 (very severe), and the total score ranges from 0 to 96, with higher scores indicating more severe symptoms [35, 36]. The test–retest reliability of the scale is 0.83, and the validity is 0.77 [37]. The study showed an MICD of 12% improvement from the baseline on this scale [38].
- (2) The pain catastrophizing scale (PCS) is a self-assessment scale used to assess the degree of exaggerated negativity in response to persistent and anticipatory pain, with a total score of 0–52; higher scores indicate more severe pain catastrophically [39].
- (3) Association of pain severity with task-state functional connectivity fNIRS. Data collection: firstly, we need to obtain the functional connectivity fNIRS in the resting state, the participants need to sit on the chair on the side of the quiet corridor, and face the wall to keep quiet and calm breathing. In this resting state, the participants wear the instrument and keep it for 5 min. Secondly, we need to obtain the task-state functional connectivity fNIRS. The participants stand up from the chair and breathe calmly for 30 s. After getting the initial instructions, the participants perform a 6-min walking test in a quiet corridor [34]. Before the start of the whole test, the test process will be explained and answered by the participants. Participants need to be quiet throughout the test.

### Sample size

The sample size will be determined by comparing the NRS scores before and after treatment based on a preliminary study of the first 10 participants. With a yielding power of 90% and a significance level of 0.05, using G*Power (3.1.9.4) software, the sample size is intended to consist of 30 participants in each group. With a 20% dropout rate, the two groups required 72 participants. KOA patients and healthy volunteers will be calculated in a ratio of 3:1, for a total of 24 healthy volunteers.

### Ethics, clinical registries, potential conflicts of interest

The trial will be conducted in accordance with the Declaration of Helsinki. The trial protocol was approved by the Biomedical Ethics Committee of West China Hospital, Sichuan University (ethics reference: 2022 (1070)).

The study was registered on the Chinese Registry website (registered in ChiCTR.org with the identifier ChiCTR2200064175).

The authors declare no conflicts of interest.

### Randomization

After the baseline measurement, the computer software SPSS (26.0) will be used to generate a random number table, and patients will be assigned to the CT or the DT in a ratio of 1:1 by a simple random method. Write the random number assigned to each group on a piece of paper and place it in an opaque envelope. Envelopes will be randomly assigned to participants by the independent individual not involved in the study.

### Blinding

This trial is single-blinded. Patients could not be blinded due to the treatment method, but each patient would only know their trial protocol at randomization. Before the start of the trial, standardized training will be given to the researchers. After grouping, an independent person will inform the researchers of the grouping, and the researchers only know the patients in this group. Evaluators, statisticians and data managers will be blinded during the trial.

### Withdrawal and adverse events

Each group has the possibility of patients dropping out of the trial. The researcher needs to record the information about the trial withdrawal, including the reason and time of withdrawal. At the same time, the researchers will record the adverse events that occurs during the trial in the CRF form, including the type, frequency, treatment measures and results of the adverse events. The adverse events will be adjudicated by three rehabilitation physicians.

## Statistical analysis

Data analysis will use an intention-to-treat analysis strategy. Data analyses will use SPSS (26.0). P ≤ 0.05 will be significantly different. The raw data collected by fNIRS will be preprocessed by NIRSPARK software, including artifact processing, filtering, segmentation and baseline comparison. At the same time, the fNIRS data will be analyzed using the statistical analysis module of MATLAB (R2020a). Component comparability of baseline data between the two groups will be performed using the chi-square test and independent samples t test. The frequency and percentage of count data will be calculated in the statistical description; the data of measurement data following a normal distribution will be described by the mean ± standard deviation. For the comparison between groups after the intervention, the independent samples t test will be used if the data follow a normal distribution, and the Mann‒Whitney U rank sum test will be used if the data are nonnormally distributed. For intragroup comparisons before and after the intervention, a paired t test will be used if the data follow a normal distribution, and the Wilcoxon rank sum test will be used for data that are not normally distributed.

## Conclusion

KOA is a chronic degenerative joint disease that causes joint pain and dysfunction. The main objective of KOA treatment is to reduce pain and improve dysfunction, in which physical factor treatment is an important intervention [40]. In this study, changes in cerebral cortical blood flow measured by fNIRS were used to evaluate changes in brain activation status in normal subjects and KOA patients and the consistency with the severity of pain in KOA patients. In addition, we will evaluate the effect of physical factor therapy on pain and dysfunction in KOA patients based on fNIRS.

The study will be conducted in the Rehabilitation Medical Center of West China Hospital of Sichuan University, and patients will be enrolled through poster recruitment and outpatient registration. This study is a noninvasive, adaptive and innovative way to evaluate the effectiveness of physical therapy in KOA patients.

FNIRS is an innovative neuroimaging technique for measuring hemodynamic changes during brain activation [41]. fNIRS has the characteristics of portability, wireless nature and lightweight, which can be used as an objective index to evaluate changes in pain [42]. A recent study used fNIRS to evaluate the effect of exercise on the severity of pain, and the results showed that there was a significant difference in the activation of the prefrontal cortex before and after exercise [22]. However, there is currently no study comparing changes in cerebral hemodynamics between healthy subjects and KOA patients and whether changes in cerebral hemodynamics are used as objective indicators of pain and functional improvement in KOA patients.

Therefore, the advantages of this study are mainly summarized as follows: (1) On the basis of previous studies, healthy subjects were added to evaluate the degree of cerebral cortex activation in healthy subjects; (2) evaluating the changes in cerebral blood flow between KOA patients with different pain severities and to provide an experimental basis for fNIRS as an objective index for the clinical pain evaluation of KOA patients; and (3) After using physical factors to treat KOA patients, we compared the changes in cerebral blood flow, NRS, and functional activity scores between different groups of KOA patients under different action instructions.

However, there are still some limitations in the design of this study: (1) This study has not yet conducted a follow-up survey, so it cannot evaluate the long-term therapeutic effect of physical factor therapy on pain and dysfunction in KOA patients; (2) In view of the nature of physical therapy in clinical research, it is difficult to achieve a double-blind experimental process (the therapist blind method and the subject blind method). In conclusion, the results of this study provide an important objective evaluation index for physical factor therapy to improve the dysfunction of KOA patients and extend a new perspective for fNIRS as a new objective clinical diagnostic index for KOA.

## Data Availability

The datasets used and/or analyzed during the current study are available from the corresponding author on reasonable request.

## Abbreviations

KOA: Knee osteoarthritis
fNIRS: Functional near-infrared spectroscopy
DT: Drug therapy group
CT: Combination therapy group
NRS: Numerical rating scale
WOMAC: Western Ontario and McMaster Universities Osteoarthritis Index
PCS: Pain catastrophizing scale
OA: Osteoarthritis
QoL: Quality of life
LIPUS: Low-intensity pulsed ultrasound
tDCS: Transcranial direct current stimulation
RCT: Randomized controlled trial
MICD: Minimum clinically important difference
M1: Motor Cortex
SMA: Supplementary motor area
tDCS: Transcranial direct current stimulation
